# Partial response to cinacalcet treatment in a patient with secondary hyperparathyroidism undergoing hemodialysis: a case report

**DOI:** 10.1186/1752-1947-6-417

**Published:** 2012-12-11

**Authors:** Giovanni Conzo, Alessandra F Perna, Salvatore Napolitano, Claudio Mauriello, Claudio Gambardella, Ersilia Satta, Giuseppe Ciancia, Giovanbattista Capasso, Luigi Santini

**Affiliations:** 1 Department of Anesthesiology, Surgical and Emergency Science, VII Division of General and Endocrine Surgery, Second University of Naples, Via Gen. G. Orsini 42, Napoli, 80132, Italy; 2 Department of Cardio-thoracic and Respiratory Sciences, First Division of Nephrology, Second University of Naples, Naples, Italy; 3 Department of Biomorphology and Functional Sciences, “Federico II” University of Naples, Naples, Italy

## Abstract

**Introduction:**

In the treatment of secondary hyperparathyroidism of chronic kidney disease, calcimimetics - allosteric modulators of the calcium-sensing receptor - inhibit glandular hyperplasia and significantly reduce circulating parathyroid hormone levels. They have a major impact on the management of secondary hyperparathyroidism.

**Case presentation:**

We present the clinical case of a 41-year-old Caucasian man undergoing chronic hemodialysis, who had a parathyroidectomy to treat severe secondary hyperparathyroidism resistant to cinacalcet treatment. Preoperatively, 24 months after high-dose cinacalcet hydrochloride, we observed a persistently elevated intact parathyroid hormone serum level, and detected clear parathyroid gland hyperplasia regression on ultrasound. We performed a three-gland parathyroidectomy, which was assumed to be total, associated with a hemithyroidectomy. Our patient then entered a hypoparathyroid state. A histopathological examination showed that the removed parathyroid glands were of small size, with a total weight of 1g, associated with a multifocal small papillary thyroid cancer.

**Conclusion:**

In the management of secondary hyperparathyroidism, cinacalcet hydrochloride effectively reduces total parathyroid gland hyperplasia. However, a persisting elevated intact parathyroid hormone serum level may be observed, demonstrating that reduced parathyroid hyperplastic tissue may still be associated with severe secondary hyperparathyroidism. Even if calcimimetics are very effective in secondary hyperparathyroidism treatment, further studies are necessary for a better understanding of their actions.

## Introduction

Secondary hyperparathyroidism (2HPT) of chronic kidney disease has a negative effect on patient morbidity, inducing a higher mortality rate, particularly due to cardiovascular complications. In patients undergoing hemodialysis (HD), the incidence of 2HPT increases with dialysis ‘vintage’. Prior to the calcimimetic era, a parathyroidectomy (PTx) was necessary in 15% of cases after 10 years, rising to 38% after 20 years [1]. First marketed in 2004, calcimimetics - allosteric modulators of the calcium-sensing receptor - inhibit glandular hyperplasia and significantly reduce circulating parathyroid hormone (PTH) levels [2]. Before their introduction, standard medical treatment, consisting of phosphate chelators (calcium carbonate, sevelamer, lanthanum carbonate), dialysis baths with various calcium concentrations, and vitamin D and its analogues, was ineffective in many patients. Calcimimetics have demonstrated a major impact on 2HPT management. Nevertheless, nodular hyperplasia, typically associated with more advanced 2HPT, is less responsive to conservative treatment, presumably because of the reduced expression of calcium-sensing receptors (CaSR) and vitamin D receptors (VDR). Although cinacalcet hydrochloride effectively reduces intact parathyroid hormone (iPTH) levels, and is also effective in patients refractory to vitamin D therapy, the effect of cinacalcet hydrochloride in marked glandular hyperplasia is still the subject of active research [3]. We report the case of a patient with 2HPT, documenting that cinacalcet hydrochloride is able to reduce gland size, but is not able in all cases to reduce PTH secretion.

## Case presentation

We observed a 41-year-old Caucasian male patient who underwent, at the age of 10 years, resection of epileptogenic tubers for tuberous sclerosis. He was also treated with anticonvulsants for postoperative secondary refractory epilepsy (last therapy: phenobarbital 200mg per day; levetiracetam 1000mg per day; lamotrigine 150mg per day; vigabatrin 1500mg per day). He had giant kidney angiomyolipomas and polycystic kidney disease. At 35 years old, he was started on standard three-weekly HD treatment required for end-stage renal disease. A bilateral nephrectomy was then performed.

Our patient developed severe 2HPT, and was managed by paricalcitol (Zemplar®, 5μg three times a week) and sevelamer (Renvela®, 7.2g per day). Subsequently, he developed acute alcoholic pancreatitis, and during this period his compliance to therapy was insufficient. In addition, he was ineligible for transplantation because of his severe comorbidities. His 2HPT became resistant to medical treatment and was associated with an iPTH serum level of 1123pg/mL (normal range 4.6 to 58.1pg/mL), serum calcium of 7.3mg/dL (normal range 8.9 to 10.3mg/dL, value corrected with serum albumin), serum phosphate (P) of 4.7mg/dL (normal range 2.4 to 4.1mg/dL) and 25-hydroxy vitamin D of 15ng/mL (normal range 30 to 74ng/mL) (Table 1). Cinacalcet hydrochloride (30mg per day) was administered from January 2010, and the dose was titrated upwards in the following period. In March 2011, his iPTH level was 1367pg/mL, and a cinacalcet hydrochloride dosage of 180mg per day was required. Our patient was referred to surgery for 2HPT resistant to medical treatment, associated with persistently elevated serum iPTH levels and clinical symptoms - anemia resistant to erythropoiesis-stimulating agent therapy, arthromyalgias, osteopenia and mood alterations. However, due to several intervening complications (arteriovenous fistula closure, catheter infection), surgery was postponed and performed in February 2012.

**Table 1:** Mean monthly measurements performed during the year

	**2010**	**2011**	**2012**
Parathyroid hormone (pg/mL)	1123	1367	848
Calcium (mg/dL)	7.3	7.9	7.5
Phosphate (mg/dL)	4.7	5.4	5.5
Alkaline phosphatase (U/L)	770	678	700
Hemoglobin (g/dL)	10.0	9.2	9.0
Cinacalcet dose (mg/day)	30	90 to 180	180
Paricalcitol (mg/week)	15	15	15
Sevelamer (mg/day)	4.8	7.2	7.2
Erythropoietin beta (UI)	3000×3	6000×2	6000×3

Of note, asymptomatic hypocalcaemia is present as well as anemia resistant to erythropoietin administration, requiring the increase of the erythropoietin dose (the patient was iron replete).

Preoperative instrumental work-up had consisted of measurements of his iPTH, calcium, phosphate, alkaline phosphatase, free tri-iodothyronine, free thyroxine, thyroid-stimulating hormone and thyroglobulin serum levels; high resolution neck ultrasound (US); ear, nose and throat examination; and technetium-99m sestamibi scintigraphy. The LIAISON® intact PTH Assay (DiaSorin Inc., Stillwater, MN, USA), based on a chemiluminescence immunoassay, was used for the quantitative determination of iPTH. A cardiac evaluation included a 12-lead echocardiogram and epiaortic two-dimensional and color Doppler transthoracic echocardiogram. US examination showed two bilateral hypoechoic hyperplastic parathyroid glands, of 2.8×1.5×1.5cm (right gland) and 2.3×1.3×1.4cm (left gland). A technetium-99 sestamibi scintigraphy revealed hyperactive uptake activity in the same areas. Twelve months after initiating cinacalcet hydrochloride therapy, a regression of the parathyroid gland hyperplasia was detected by US (right gland: 2.2×1.0×0.9cm; left gland: 1.9×1.0×0.7cm).

After an extensive neck exploration, including comprehensive exploration of the thyrothymic ligament, bilateral thymic tongues and the jugular-carotid axis to rule out the presence of ectopic and supernumerary glands, only three glands were removed. Extemporary histological examination confirmed the nature of these removed glands. We performed a, presumed total, PTx, not associated with autoimplantation. We also carried out a right loboisthmusectomy associated with thyrothymic ligament resection to look for an intrathyroidal parathyroid gland. Parathyroid tissue was not cryopreserved for re-transplant.

**Figure 1:**
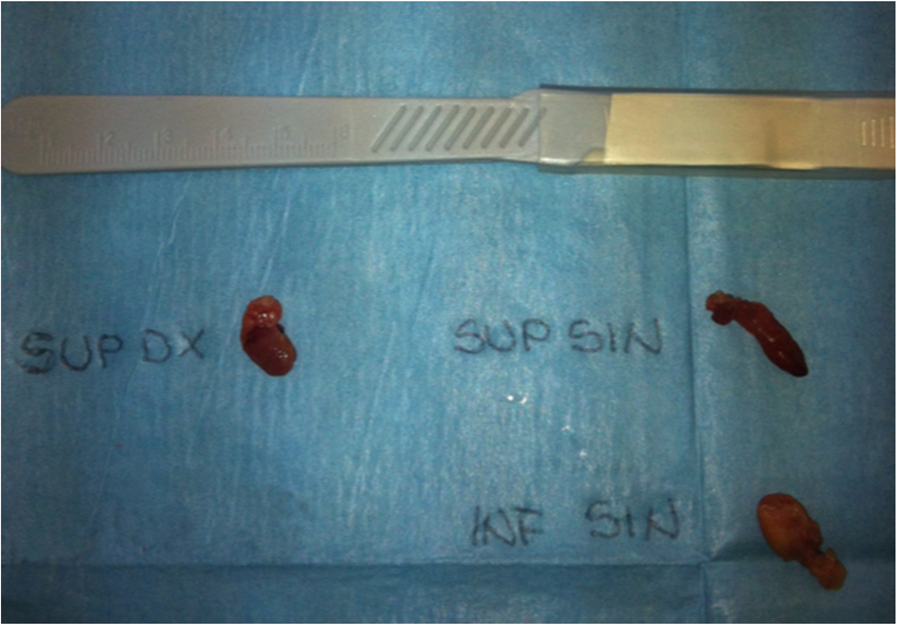
**Removed parathyroid glands.** Left lower parathyroid gland: 1.2×0.8×0.3cm, weight 0.5g; left upper parathyroid gland: 1.7×0.5×0.2cm, weight 0.3g; right upper parathyroid gland: 0.7×0.6×0.3cm, weight 0.2g.

Our patient’s iPTH serum level was 629pg/mL before anesthesia induction, 45pg/mL 20 minutes after surgery, and 4pg/mL 24 hours after surgery. Severe hypocalcemia was observed postoperatively, which was, however, never associated with hypocalcemic seizures. A pathological examination showed three hyperplastic nodular parathyroid glands and a multifocal small papillary thyroid carcinoma. The macroscopic parathyroid gland features are reported in Figure 1. Parathyroid tissue showed diffuse hyperplasia of chief cells with minimal fat. A focally nodular pattern was observed, while infarction, and fibrous or cystic degeneration were not observed. In order to assess the degree of apoptosis in the examined parathyroid tissue, an immunohistochemical study with the B-cell lymphoma-2 protein was performed, with adequate controls, using a monoclonal antibody, clone 124-Ventana® (Ventana Medical Systems, Inc., Tucson, AZ, USA). No B-cell lymphoma-2 positive-staining cells were detected in any of the examined glands.

Our patient’s postoperative course was uneventful and he was discharged on day 4. Hypoparathyroidism (iPTH <4pg/mL) associated with persisting severe hypocalcemia was recorded two weeks and one month after surgery. Two months postoperatively, our patient had a subarachnoid hemorrhage and was moved to our intensive unit care in a comatose status. Fourteen days later, after recovery to generally acceptable clinical conditions, our patient was discharged.

## Discussion

In 2HPT of chronic kidney disease, parathyroid gland hyperplasia, especially in its nodular form, is associated with reduced expression of CaSR and VDR, determining medical therapy resistance. Before cinacalcet hydrochloride was introduced, calcitriol and other vitamin D analogues, which also enhance calcium and phosphate serum levels, were considered the cornerstone of medical therapy. A glandular volume greater than 500mm^3^ and nodular hyperplasia, often observed in patients refractory to vitamin D treatment, are still considered the main medical treatment resistance factors. Subsequently, it has been shown that cinacalcet hydrochloride effectively reduces iPTH levels while maintaining calcium and phosphate levels within the normal range [3]. Komaba [4] reported calcium treatment efficacy, confirming a rapid decline in serum PTH levels, by 50%, in patients affected by severe 2HPT associated with suspected nodular hyperplasia and reduced expression of CaSR and VDR. Cinacalcet hydrochloride therapy has been shown to upregulate VDR and CaSR expression, facilitating medical therapy outcomes. Experiments carried out in animal models demonstrated reduced vascular and extraosseous calcifications.

Cinacalcet hydrochloride effects on the parathyroid gland may also result in cell hypertrophy reduction and increased apoptosis, determining the decrease in iPTH serum levels. Its use may also increase oxyphil to chief cell ratios and cystic degeneration. According to literature data [4-6], a decrease in PTH serum level is considered the main effect of cinacalcet hydrochloride therapy, whereas parathyroid gland hypertrophy reduction is not reported in all patients. On the contrary, in the reported case, despite high-dose cinacalcet hydrochloride therapy (180mg/day), carried out for a significant amount of time (over six months), a significant reduction in glandular volume, associated with persisting high iPTH serum level, was observed on US. Only after PTx did a hypoparathyroid state with severe hypocalcemia follow. However, we have to consider that prolonged therapy with anticonvulsants may cause vitamin D deficiency and hypocalcaemia [7].

We can consider several reasons for the low calcium levels observed in the face of high iPTH: a small amount of VDR expression in the cells; low compliance to oral calcium therapy; and drug interaction by anticonvulsants. In addition, other factors besides CaSR and VDR activity influence PTH release and synthesis. In particular, the latter is regulated by *PTH* and *CaSR* gene transcription, and variations in the amounts of messenger ribonucleic acid (mRNA)-encoding *PTH*-binding proteins, which are important for mRNA stability, can occur. RNA-binding proteins interact with sequence-specific elements, adenine- and uridine-rich elements, regulating the rate at which mRNAs are translated or degraded [8]. In addition, fibroblast growth factor 23 (FGF-23), usually much increased in patients on HD, has direct actions on the parathyroid gland, where it suppresses PTH secretion [9]. A markedly decreased expression of FGF receptor 1 and Klotho protein (a transmembrane protein required for FGF-23 receptor activation) in a hyperplastic parathyroid gland is present in patients on HD [10]. It is possible to hypothesize that variations of one of these factors are at play in explaining the reason that high PTH levels were present in our patient in association with a reduction in gland size.

## Conclusions

Our data suggest that further studies are necessary for a better understanding of the actions of cinacalcet hydrochloride that cause its pharmacological effects. In particular, after cinacalcet therapy, we observed an unusual small gland volume compared with other reported cases of 2HPT, while iPTH levels remained elevated. A few considerations can be made: first, in patients with 2HPT and persistently elevated iPTH serum levels after six months of high-dosage cinacalcet hydrochloride, medical resistance is present and surgical intervention should be recommended, especially in young patients. Second, regarding surgical treatment, a <10% intraoperative decrease of iPTH with respect to the baseline value, 20 minutes after PTx, may be considered predictive of total PTx [1]. Therefore, we suggest that autoimplantation is a better choice in patients on a waiting list for renal transplantation, especially when only three parathyroid glands are intraoperatively identified and removed [11]. In fact, it is well recognized that in only about 3% of patients with 2HPT on HD will three glands be present [1]. The third consideration is that, in cases of unresponsiveness to medical treatment for 2HPT, especially in young patients on HD, a multidisciplinary, early, aggressive treatment (early surgery or high-dose calcimimetics therapy) must be recommended to avoid severe cardiovascular events and other complications related to chronic kidney disease, and to ameliorate quality of life.

## Consent

Written informed consent was obtained from the patient for publication of this case report and accompanying images. A copy of the written consent is available for review by the Editor-in-Chief of this journal.

## Competing interests

The authors declare that they have no competing interests.

## Authors’ contributions

GC and AFP analyzed and interpreted the patient’s data and were major contributors in writing the manuscript. GC undertook pathological evaluation of the parathyroid glands. All authors read and approved the final manuscript.
